# RACK1 Associates With STING to Promote Type I Interferon Activation and Inhibit Pseudorabies Virus Infection

**DOI:** 10.1155/tbed/9584967

**Published:** 2025-12-11

**Authors:** Yixuan Li, Yiyu Liu, Yu Dai, Jingyi Niu, Gang Li, Xiaoying Yu, Chao Wan, Rendong Fang, Chao Ye

**Affiliations:** ^1^ Joint International Research Laboratory of Animal Health and Animal Food Safety, College of Veterinary Medicine, Southwest University, Chongqing, 400715, China, swu.edu.cn

**Keywords:** pseudorabies virus, RACK1, STING, type I interferon

## Abstract

Pseudorabies virus (PRV), the causative agent of Aujeszky’s disease, leads to great economic losses on swine production worldwide. Receptor of activated protein kinase C 1 (RACK1) is initially known as a receptor for protein kinase C, and recent studies indicate that RACK1 can also play critical roles in various virus infections. However, the role of RACK1 during PRV infection has not yet been determined. In this investigation, we observed a strong positive correlation between the expression levels of RACK1, interferon‐β (IFN‐β), and the IFN‐stimulated gene 15 (ISG15) and ISG20 in PRV‐infected porcine kidney‐15 (PK‐15) cells at 24 h postinfection. Further experiments revealed that RACK1 exerted an inhibitory effect on PRV replication and enhanced the activation of the Type I IFN (IFN‐I) signaling pathway. Interestingly, RACK1 was found to facilitate stimulator of IFN genes (STING)–dependent phosphorylation of IFN regulatory factor 3 (IRF3). More specifically, RACK1 could interact with STING and then promote aggregation of STING around the Golgi apparatus. Taken together, these findings demonstrated that RACK1 could associate with STING to promote IFN‐I activation and inhibit PRV infection. These results will provide new data on host factors that limit PRV infection, and facilitate our understanding of IFN‐I‐mediated antiviral responses during PRV infection.

## 1. Introduction

Pseudorabies virus (PRV), an alphaherpesvirus that primarily infects swine, is responsible for Aujeszky’s disease [1]. Infection with PRV leads to lethal encephalitis and high mortality in piglets, reproductive failure in sows, and respiratory illness in adult pigs, contributing to substantial economic losses in pork production worldwide [2]. In addition, like several other members of herpesviruses, PRV has the characteristics of latent infection, and the infectious viruses can be reactivated from its latent stage by various stimuli [3]. Due to the characteristics of latent infection and pathogenicity to pigs, PRV infection in pigs is difficult to eliminate completely and has caused serious economic losses to the global pig industry [4]. In recent years, several studies have also indicated that PRV can infect humans and cause meningitis [5–8]. Although PRV has not been found to be transmitted between humans, the potential threat of PRV to public health is still of concern.

The cyclic GMP‐AMP synthase (cGAS) and stimulator of interferon (IFN) genes (STING) pathway serves as a main effector of cell sensing pathogenic double‐stranded (ds) DNAs and self‐aberrant DNAs, playing a critical role in anti‐infection and antitumor immune responses [9]. In response to infection, cGAS can recognize pathogenic dsDNAs and then induce the production of second messenger cyclic GMP‐AMP (cGAMP), which subsequently activates STING located on the endoplasmic reticulum. After its activation, STING can be transferred from the endoplasmic reticulum to the Golgi apparatus, and further triggers the Type I IFN (IFN‐I) responses through the activation of TANK‐binding kinase 1 (TBK‐1) and IFN regulatory factor 3 (IRF3) [10]. As a dsDNA virus, PRV infection can activate cGAS–STING–IRF3 axis and promote the expression of IFN‐β [11]. Moreover, several studies indicated that PRV could also inhibit this pathway through a variety of mechanisms [12–14]. However, the potential involvement of additional host molecules in activation of cGAS–STING pathway during PRV infection remains largely unexplored.

Receptor of activated protein kinase C 1 (RACK1) is a multifunctional scaffold protein implicated in diverse cellular processes, including cell proliferation, apoptosis, protein translation, and neural signaling. Emerging evidences also indicated its involvement in the modulation of viral infections [15–17]. Studies showed that RACK1 could function as a significant host regulator during viral infection, exhibiting divergent roles across different viruses. It has been found to facilitate the replication of lymphocystis disease virus (LCDV), porcine reproductive and respiratory syndrome virus (PRRSV), dengue virus (DENV), and hepatitis C virus (HCV). In contrast, RACK1 could inhibit the proliferation of classical swine fever virus (CSFV) in porcine kidney‐15 (PK‐15) cells [18–23]. Furthermore, RACK1 could also regulate several innate immune pathways during viral infections, such as the IFN‐I signaling pathway and the NF‐κB signaling pathway [21, 24, 25]. Cao et al. [26] also found that the matrix protein of respiratory syncytial virus could suppress IFN signaling via association with RACK1. However, the effect of RACK1 upon regulating viral proliferation and innate immune pathways during PRV infection is still unclear.

In this study, we reported that RACK1 could enhance host defense against PRV infection in host cells via promoting the activation of IFN‐I pathway. Furthermore, our study unraveled a role of RACK1 in mediating IFN‐I activation via association with STING. Taken together, we uncovered a novel role of RACK1 in regulating IFN‐I response during PRV infection, which enhances our understanding of the host innate immune responses during PRV infection.

## 2. Materials and Methods

### 2.1. Virus and Cells

The PK‐15 cells, African green monkey kidney (Vero) cells, baby hamster kidney‐21 (BHK‐21) cells, and human embryonic kidney (HEK‐293T) cells were maintained in DMEM containing 10% fetal bovine serum (FBS), 100 U/mL penicillin, and 100 μg/mL streptomycin, and incubated at 37°C under 5% CO_2_ in a humidified atmosphere. The PRV strain JS‐2012, a PRV variant, was propagated and titrated in Vero cells. Virus stocks were stored at −80°C. Viral titers were determined by TCID_50_ assay on Vero cells using the Reed–Muench method [27].

### 2.2. Antibodies

The following primary antibodies were employed: anti‐PRV glycoprotein E (gE) antibody (Ab; preserved by our lab), anti‐RACK1 Ab (Cell Signaling Technology, USA), anti‐phospho‐IRF3 Ab (Cell Signaling Technology, USA), anti‐STING Ab (Proteintech, China), anti‐IRF3 Ab (Beyotime, China), anti‐β‐actin Ab (Beyotime, China), mouse anti‐Flag tag Ab (Cell Signaling Technology, USA), rabbit anti‐Flag tag Ab (Sigma Aldrich, USA), mouse anti‐HA tag Ab (Proteintech, China), rabbit anti‐HA tag Ab (Sigma Aldrich, USA), CoraLite 594‐conjugated RACK1 (Proteintech, China), and anti‐Golgin‐97 Ab (Thermo Fisher Scientific, USA).

### 2.3. RNA Interference

All siRNAs applied in this study, whose sequences were provided in Table 1, were synthesized by Sangon Biotech (Shanghai, China). For transfection, PK‐15 cells were first plated in 12‐well plates. Upon reaching 70%–80% confluence, the cells were transfected with either control siRNA (si‐Control) or RACK1 siRNA (si‐RACK1) at a final concentration of 50 nM, using Lipofectamine RNAiMAX Transfection Reagent (Thermo Fisher Scientific, USA). At 36 h after transfection, the transfected cells were either infected with PRV or stimulated with 2^′^,3^′^‐cGAMP for specified durations, and then the cells were collected for subsequent quantitative RT‐PCR (qRT‐PCR) and western blot analyses.

**Table 1:** Sequences of siRNA.

Name	Sense (5′–3′)	Antisense (5′–3′)
si‐Control	UUCUCCGAACGUGUCACGUTT	ACGUGACACGUUCGGAGAATT
si‐RACK1	CCAUCAAGCUAUGGAAUACTT	GUAUUCCAUAGCUUGAUGGTT

### 2.4. Plasmids Construction and Transfection

The porcine RACK1 open reading frame (ORF) was obtained by reverse transcription of total RNA isolated from PK‐15 cells and PCR amplification using the corresponding primers listed in Table 2. Then, the resulting product was cloned into the pMD19‐T vector (TaKaRa, China) and confirmed by Sanger sequencing. Subsequently, the RACK1 insert was subcloned into the eukaryotic expression vector pcDNA3.1(+) and p3 × FLAG‐CMV, respectively. The plasmid‐encoding porcine STING with an HA tag (pCAGGS‐STING‐HA), previously constructed and preserved in our lab, was also used in this study.

**Table 2:** Sequences of primers for plasmid construction.

Primer name	Sense (5′–3′)	Antisense (5′–3′)
RACK1	CGCCACCATGACTGAGCAGAT	CTAGCGCGTGCCGATGGT
RACK1‐Flag	CGAAGCTTATGACTGAGCAGAT	CGGAATTCCTAGCGGGTGCCAATGGT

To assay for overexpression of RACK1, PK‐15 cells were seeded onto 12‐well plates (~2 × 10^5^ cells per well) and then treated with the transfection complex containing TransIT‐X2 Transfection Reagent (Mirus, USA) and the overexpression vector of RACK1 (pcDNA3.1‐RACK1/p3 × FLAG‐CMV‐RACK1) or empty plasmid (pcDNA3.1(+)/p3 × FLAG‐CMV) for 48 h. Subsequently, the PK‐15 cells were infected with PRV JS‐2012 or stimulated with 2^′^,3^′^‐cGAMP (MedChemExpress, China) for the indicated time. Cells transfected with empty vector and cells without PRV infection/2^′^,3^′^‐cGAMP treatment were used as controls.

### 2.5. Western Blot Analysis

Whole‐cell lysates were prepared using RIPA lysis buffer (Beyotime, China) supplemented with PMSF protease inhibitor (Beyotime, China), SDS loading buffer (Beyotime, China), and PhosSTOP phosphatase inhibitor (Roche, USA). Proteins were separated by 12% SDS‐PAGE and transferred to PVDF membranes. After blocking with 5% (*w*/*v*) nonfat dehydrated milk or BSA in TBST, the membranes were probed with the designated antibodies. Protein bands were visualized by ECL reagent (Biosharp, China), and their intensities were quantified using Image J software.

### 2.6. qRT‐PCR Analysis

Total RNA was isolated from cells using TRIzol Reagent (Life Technologies Carlsbad, USA) following the manufacturer’s protocol. cDNA was synthesized using the Evo M‐MLV RT Kit with gDNA Clean for qPCR (Accurate Biotechnology, China). The mRNA transcription level of the corresponding gene was determined by qRT‐PCR with the primer pairs listed in Table 3. The reaction was performed using 2 × Universal SYBR green fast qPCR mix reagent (ABclonal, China) on the CFX96 real‐time PCR detection system.

**Table 3:** Primer sequences for qRT‐PCR.

Name	Sense (5′–3′)	Antisense (5′–3′)
*GAPDH*	GAAGGTCGGAGTGAACGGATTT	TGGGTGGAATCATACTGGAACA
*IFN-β*	GGCTGGAATGAAACCGTCAT	TCCAGGATTGTCTCCAGGTCA
*RACK1*	TTCTCCTCTGACAACCGGCA	GCCATCCTTGCCTCCAGAA
*ISG15*	GATCGGTGTGCCTGCCTTC	CGTTGCTGCGACCCTTGT
*ISG20*	TCAGCTGTGCCAAATGCTGG	AAATGGGGCTTCTCCGGGTTTT
*Mx1*	CCACCTGAAGAAGGGCTAC	AACAGGGGCAGAGTTTTAC
*IFIT3*	GAAGGGTGACAACCAGAACGAC	AGGTTCCGACTTTGCCCTGATT

### 2.7. Immunofluorescence Assay

Cells were fixed in ice‐cold 4% paraformaldehyde (Sango Biotech, China) for 30 min, permeabilized using 0.1% Triton X‐100 in PBS for 5 min, and blocked with 5% BSA in PBS for 1 h. After PBS washing, samples were inoculated with the corresponding primary antibodies overnight at 4°C. For immunofluorescence detection of RACK1 and gE, samples were first incubated with an Alexa Fluor 488‐conjugated goat anti‐mouse IgG (H&L; Abcam, UK) at room temperature for 1 h. This was followed by incubation with a CoraLite 594‐labeled RACK1 Ab (Proteintech, China) at 4°C for 6 h. But for other targets, a combined incubation with goat anti‐mouse IgG (H&L) Alexa Fluor 488 (Abcam, UK) and goat anti‐rabbit IgG (H&L) Alexa Fluor 594 (Abcam, UK) was conducted for 1 h at room temperature. Nuclei were counterstained with DAPI (Beyotime, China) for 5 min, and then samples were mounted with antifluorescence attenuation mounting medium (Solarbio, Beijing, China). Imaging was performed using an inverted fluorescence microscope (Olympus, Japan), a Zeiss LSM 800 laser scanning confocal microscope (Zeiss, Germany), or an Olympus FV3000 confocal microscope (Olympus, Japan).

### 2.8. Co‐Immunoprecipitation (Co‐IP) Assay

The HEK‐293T cells were treated with transfection complex containing the overexpression plasmids (pCAGGS‐STING‐HA/p3 × FLAG‐CMV‐RACK1) and/or empty plasmid (pCAGGS/p3 × FLAG‐CMV) for 24 h. Subsequently, the cells were lysed in ice‐cold lysis buffer (Beyotime, China) for Western blot and Co‐IP. After cell lysis, under 4°C 12,000 rpm for 10 min, and then treated with Protein A + G Agarose (Beyotime, China) as well as the specified antibodies and the corresponding species IgG Ab incubation under 4°C overnight.

### 2.9. Structural Prediction and Protein Docking

The amino acid sequences for porcine RACK1 (accession number AAD37978.1) and STING (accession number ACJ70708.1) were retrieved from the GenBank database. The three‐dimensional structures of RACK1 and a C‐terminally truncated STING (residues 1–350) were predicted using the AlphaFold 3 algorithm with default settings via the online server (https://alphafoldserver.com/). The resulting models were subsequently downloaded and visualized with Pymol to identify the interfacial residues.

### 2.10. Statistical Analysis

Data are presented as mean ± standard deviation (SD). Group comparisons were performed using a two‐tailed unpaired Student’s *t*‐test. Significance thresholds were defined as  ^∗^ *p* < 0.05,  ^∗∗^ *p* < 0.01,  ^∗∗∗^ *p* < 0.001, and  ^∗∗∗∗^ *p* < 0.0001; ns denotes not significant.

## 3. Results

### 3.1. PRV Downregulated the Expression of RACK1, IFN‐I, and IFN‐Stimulated Genes (ISGs) During the Late Phase of its Infection

Several studies have demonstrated the critical roles of RACK1 in viral infections. These studies have found that RACK1 can be upregulated by infections with viruses such as bovine ephemeral fever virus and PRRSV [18, 25]. In contrast, RACK1 expression is downregulated during infections with other viruses, including CSFV and Epstein–Barr virus [21, 28]. To assess whether endogenous RACK1 expression is modulated following PRV infection, BHK‐21, and PK‐15 cells were either mock‐treated or infected with PRV at an MOI of 1 for different time points, and then the effect of PRV infection on cellular RACK1 expression was investigated. Initial western blot analysis showed that the protein expression level of RACK1 was downregulated in both BHK‐21 and PK‐15 cells at 24 h post infection (hpi) with PRV (Figure 1A,B). Then, we focused subsequent experiments on PK‐15 cells. Further analysis by qRT‐PCR and immunofluorescence consistently revealed a significant decrease in both RACK1 mRNA transcription and protein expression in PRV‐infected PK‐15 cells at 24 hpi (Figure 1C,D), suggesting a potential involvement of RACK1 in the host response to PRV infection.

Figure 1PRV downregulated the expression of RACK1, IFN‐I, and ISGs during the late phase of its infection. The BHK‐21 (A) and PK‐15 (B) cells were infected with PRV (MOI = 1) for the indicated time points, respectively. Then, the protein levels of RACK1 and PRV gE were detected by western blot. The protein levels of PRV gE and RACK1 were quantified by Image J. And the transcription level of RACK1 in PK‐15 cells was detected by qRT‐PCR (C). In addition, the expression of RACK1 and PRV gE (D) in PK‐15 cells infected with PRV (MOI = 1) was observed under inverted fluorescence microscope at 12 hpi or 24 hpi, respectively. At 0, 3, 6, 12, and 24 hpi, the transcriptional levels of IFN‐β (E), ISG15 (F), and ISG20 (G) in PRV‐infected PK‐15 cells were also detected by qRT‐PCR. The statistical difference is expressed as  ^∗^ *p* < 0.05,  ^∗∗^ *p* < 0.01,  ^∗∗∗^ *p* < 0.001.

**Figure:**
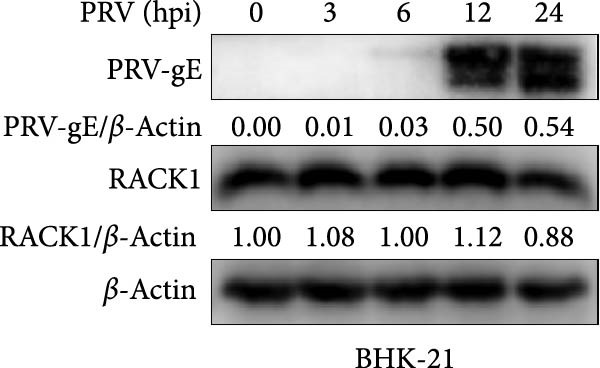
(A)

**Figure:**
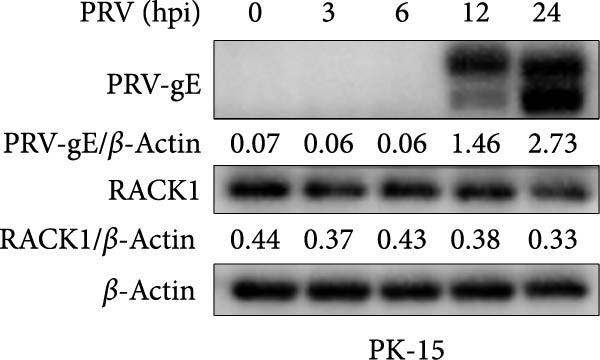
(B)

**Figure:**
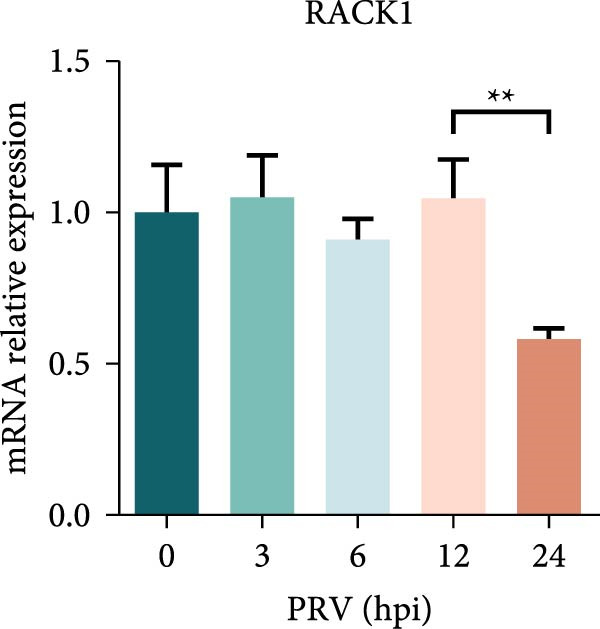
(C)

**Figure:**
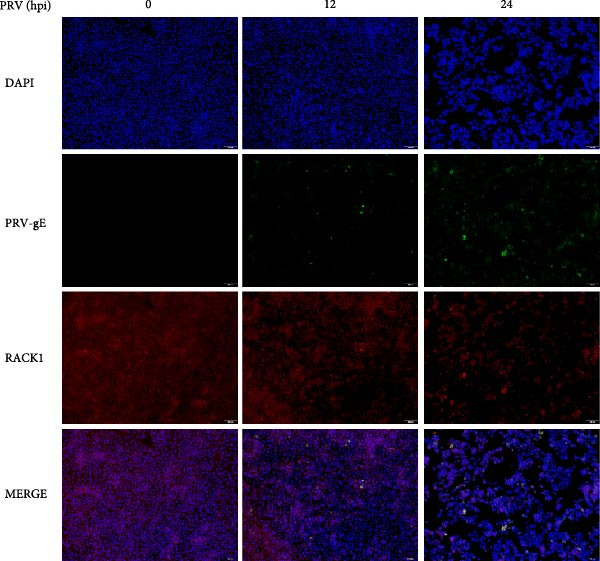
(D)

**Figure:**
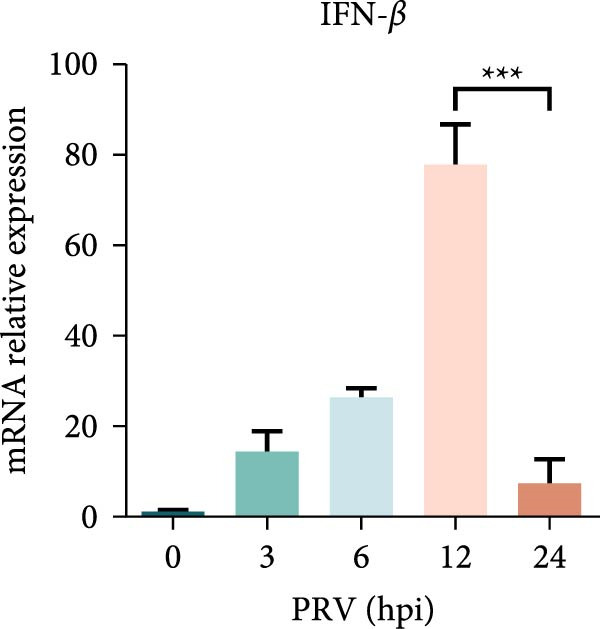
(E)

**Figure:**
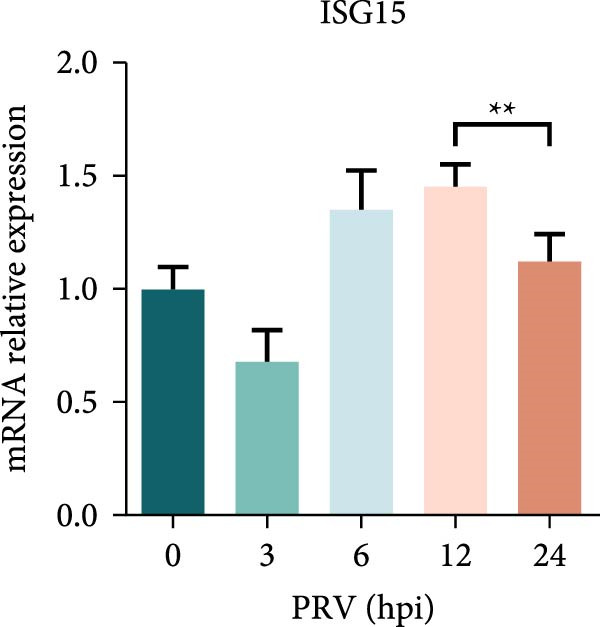
(F)

**Figure:**
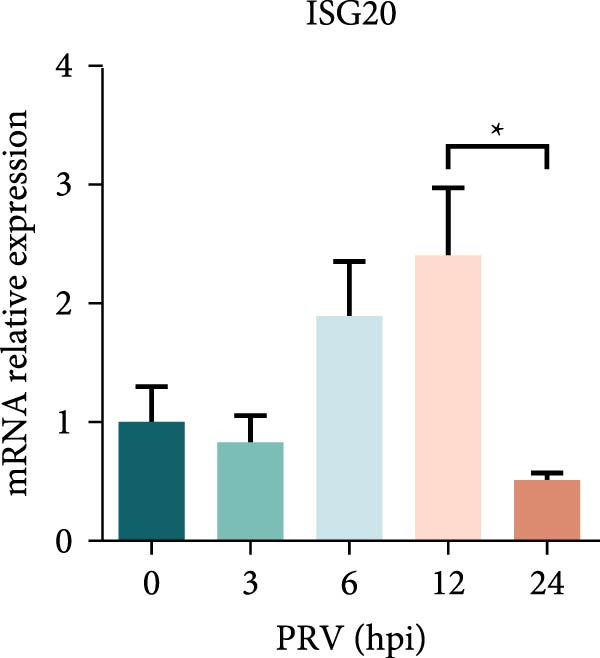
(G)

Qin et al. [29] found that RACK1 could inhibit IFN‐I production mainly by inhibiting the phosphorylation of IRF3 and IRF7 in primary macrophages, thereby affecting antiviral innate immunity. In order to explore the potential correlation between RACK1 and IFN‐I pathway during PRV infection, we measured the mRNA levels of IFN‐β, ISG15, and ISG20 in PRV‐infected PK‐15 cells using qRT‐PCR. An increase in the transcription levels of IFN‐β, ISG15, and ISG20 were observed during PRV infection till 12 hpi, followed by a significant decrease at 24 hpi (Figure 1E–G). These results indicated that PRV infection at the late phase (24 hpi) could significantly inhibit RACK1 expression and reduce IFN‐β and ISGs transcription, suggesting a potential correlation between RACK1 expression and IFN‐I pathway activation.

### 3.2. RACK1 Markedly Inhibited PRV Replication in PK‐15 Cells

Previous studies revealed that RACK1 played critical roles in regulating viral infections, which could either enhance the proliferation of LCDV and DENV or inhibit the proliferation of CSFV [20–22]. To explore whether RACK1 played a role in regulating the proliferation of PRV, RACK1 was silenced in PK‐15 cells by RNA interference. Western blot and qRT‐PCR analyses confirmed that the expression of RACK1 was significantly reduced by RACK1 interference (Figure 2A,B). Furthermore, the replication of PRV in PK‐15 cells was found increased after knockdown of RACK1, as indicated by the gE expression of PRV and the results of viral titers (Figure 2C,D).

Figure 2RACK1 inhibited PRV replication in PK‐15 cells. After treatment with small interfering RNA, the knockdown effect of RACK1 expression by si‐RACK1 was verified using western blot (A) and qRT‐PCR (B), respectively. Then, siRNA‐treated PK‐15 cells were infected with PRV (MOI = 0.1) for 24 h. Western blot was used to detect the protein expression levels of RACK1 and PRV gE in cell lysates (C), and the TCID_50_ assay was used to detect virus titer in cell supernatant (D). In addition, the overexpression effect of RACK1 in PK‐15 cells was validated using western blot (E) and qRT‐PCR (F). PK‐15 cells with RACK1 overexpressed were infected with PRV (MOI = 0.1) for 24 h, and Western blot was used to detect the protein expression levels of RACK1 and PRV gE in cell lysates (G). The TCID_50_ assay was used to detect virus titer in cell supernatant (H). The statistical difference is expressed as  ^∗^ *p* < 0.05,  ^∗∗^ *p* < 0.01.

**Figure:**
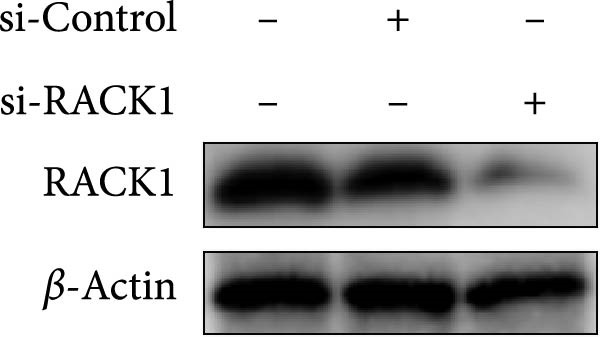
(A)

**Figure:**
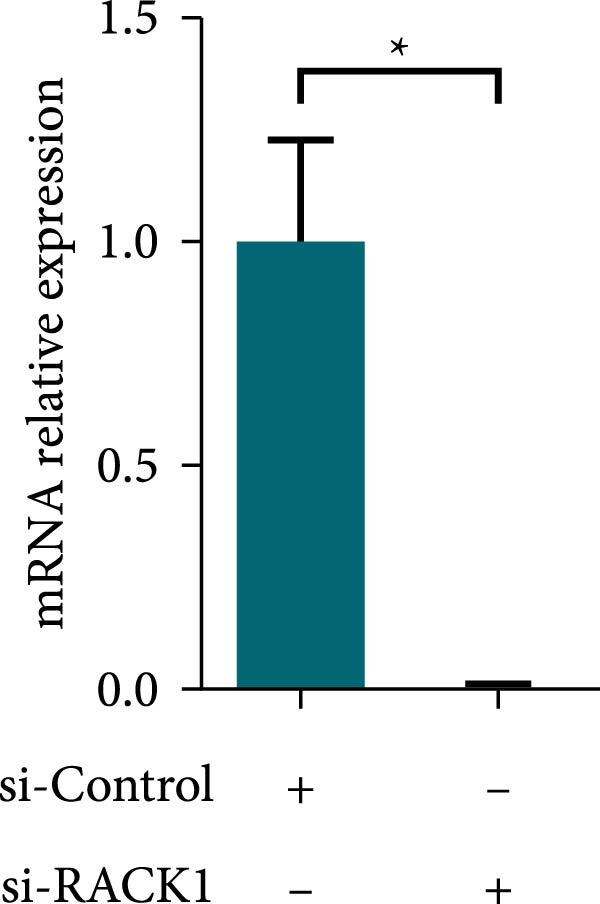
(B)

**Figure:**
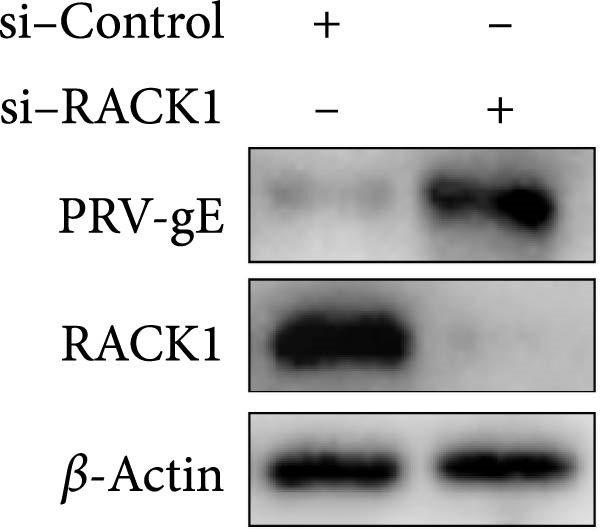
(C)

**Figure:**
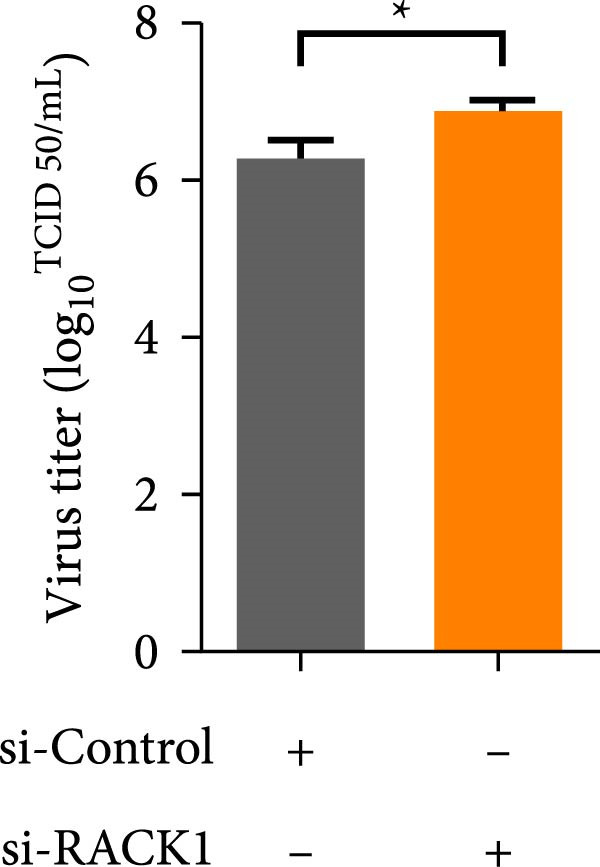
(D)

**Figure:**
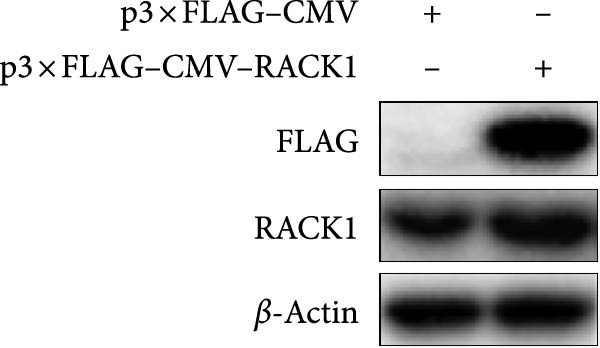
(E)

**Figure:**
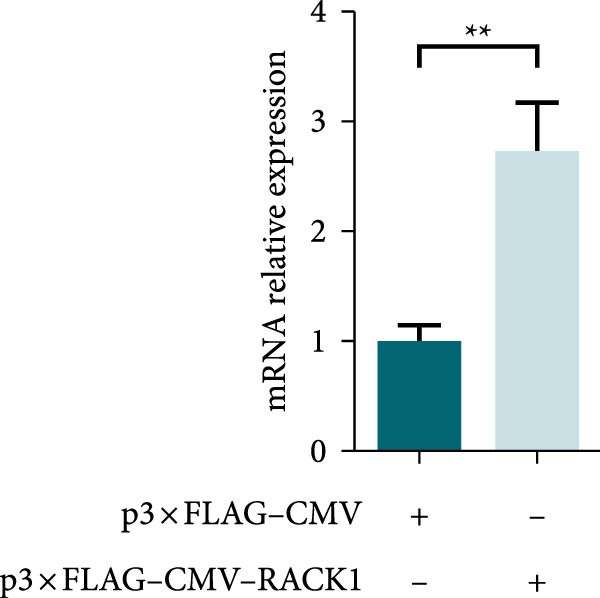
(F)

**Figure:**
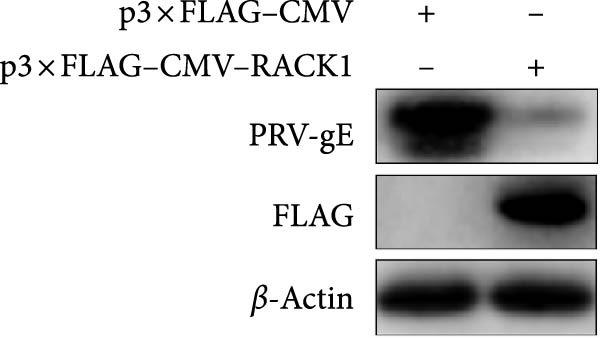
(G)

**Figure:**
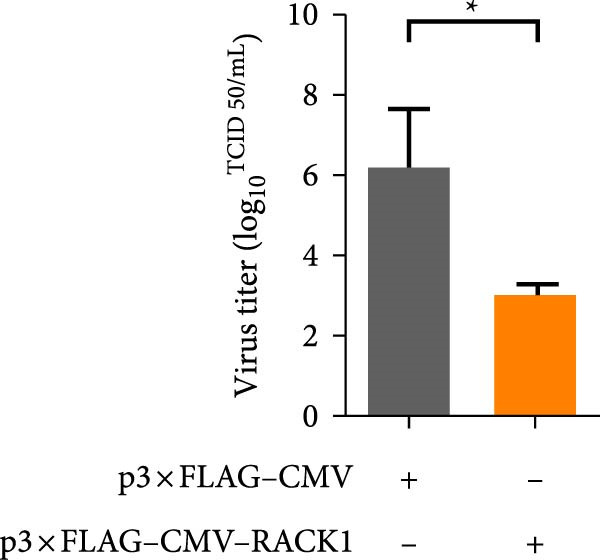
(H)

In addition, the effect of overexpression of RACK1 upon PRV replication was also investigated. First, the RACK1 overexpression was confirmed at protein and mRNA levels, as indicated by western blot and qRT‐PCR, respectively (Figure 2E,F and Figure S1). Then, the replication of PRV after RACK1 overexpression was shown significantly decreased, as supported by the results of gE expression and viral titers (Figure 2G,H and Figure S1). These findings demonstrated that RACK1 had strong inhibitory effects on PRV replication in PK‐15 cells.

### 3.3. RACK1 Enhanced the Activation of IFN‐I Pathway in Response to PRV Infection

To further investigate the potential regulatory role of RACK1 on IFN‐I pathway activation during PRV infection, the mRNA levels of RACK1, IFN‐β, ISG15, Myxovirus resistance 1 (Mx1), and IFN‐induced protein with tetratricopeptide repeats 3 (IFIT3) were measured via qRT‐PCR in PRV‐infected PK‐15 cells with RACK1 overexpressed. The results showed that RACK1 overexpression significantly upregulated the mRNA levels of IFN‐β and several ISGs, including ISG15, Mx1, and IFIT3, in PRV‐infected cells at different timepoints (6, 12, and 24 hpi; Figure 3A–E).These findings demonstrate that RACK1 enhances the expression of IFN‐β and ISGs during PRV infection, suggesting that RACK1 acts as a positive regulator of the IFN‐I signaling pathway.

Figure 3RACK1 enhanced activation of the IFN‐I pathway during PRV infection. PK‐15 cells transfected with either p3 × FLAG–CMV or p3 × FLAG–CMV–RACK1 were infected with PRV at an MOI of 1. The transcriptional levels of RACK1 (A), IFN‐β (B), ISG15 (C), Mx1 (D), and IFIT3 (E) were then analyzed by qRT‐PCR at 0, 6, 12, and 24 hpi. The statistical difference is expressed as  ^∗^ *p* < 0.05,  ^∗∗^ *p* < 0.01,  ^∗∗∗^ *p* < 0.001,  ^∗∗∗∗^ *p* < 0.0001, ns refers to not significant.

**Figure:**
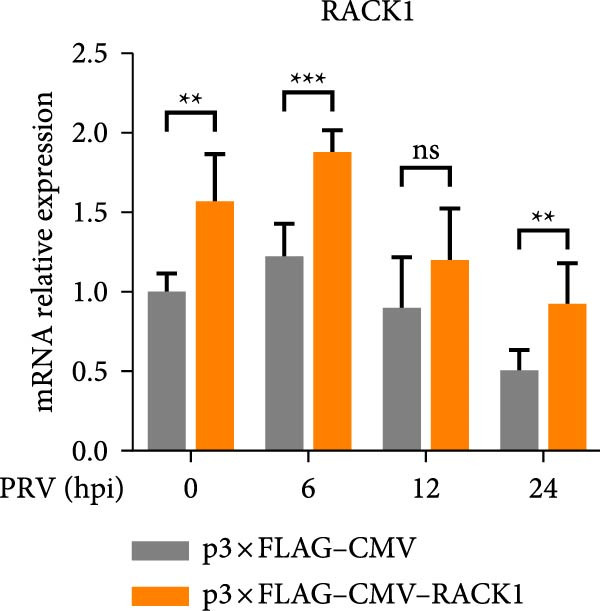
(A)

**Figure:**
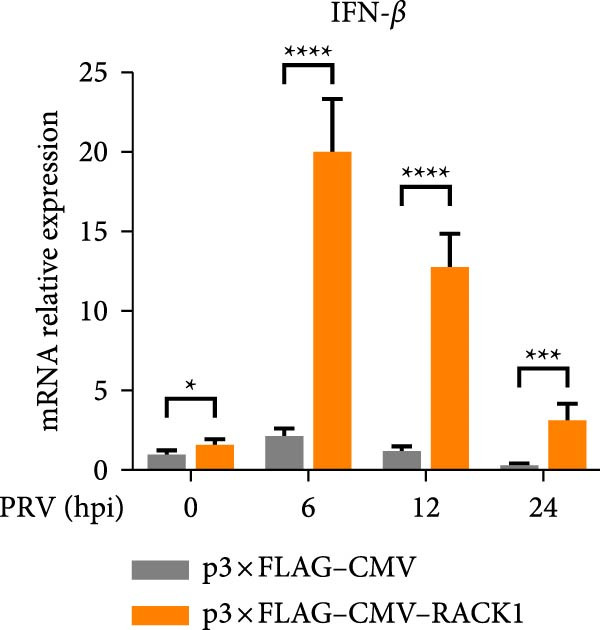
(B)

**Figure:**
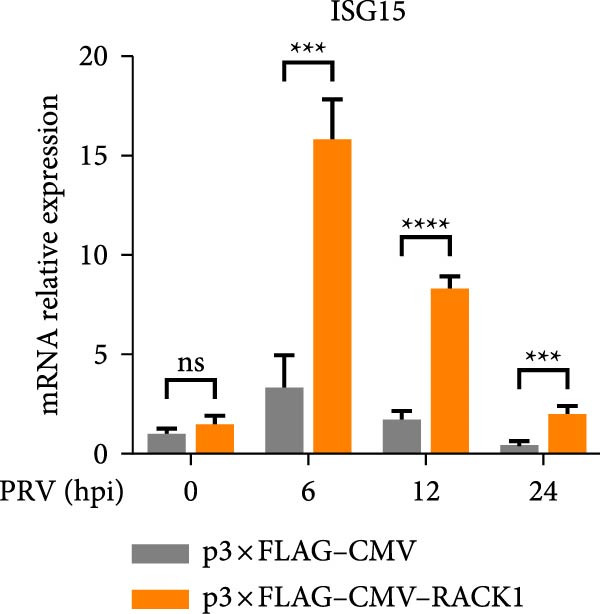
(C)

**Figure:**
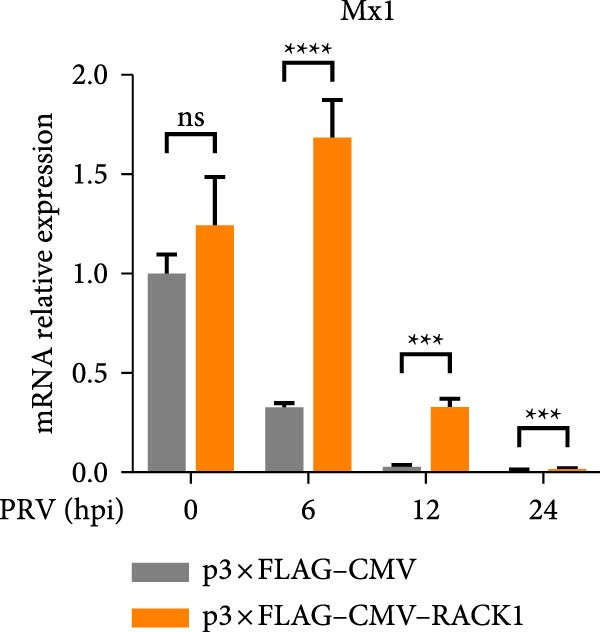
(D)

**Figure:**
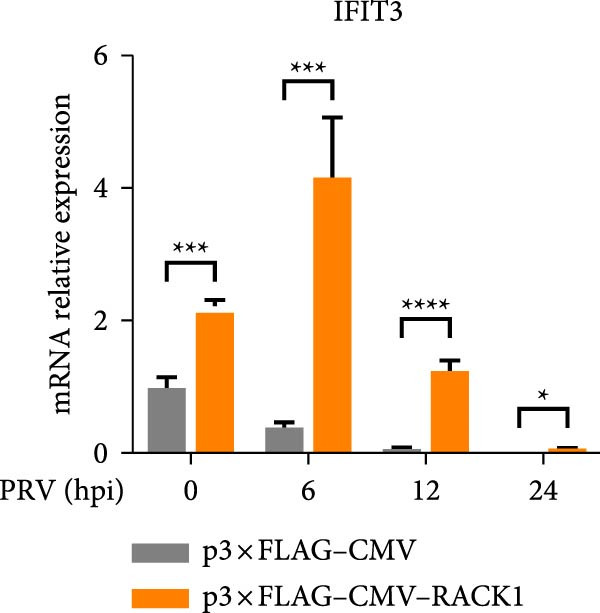
(E)

### 3.4. RACK1 Promoted STING‐Mediated Phosphorylation of IRF3

To explore how RACK1 affected activation of IFN‐I pathway during PRV infection, western blot was adopted to detect the protein expression levels of STING and p‐IRF3/IRF3 in PRV‐infected PK‐15 cell lysate samples with silencing or overexpressing RACK1. The results indicated that PRV infection induced phosphorylation of IRF3, however, no obvious difference in PRV‐induced IRF3 phosphorylation was observed between the si‐RACK1 and si‐Control groups (Figure 4A). In contrast, overexpression of RACK1 led to a noticeable increase in p‐IRF3 expression levels in PRV‐infected cells (Figure 4B).

Figure 4RACK1 promoted STING‐mediated phosphorylation of IRF3. PK‐15 cells were transfected with si‐RACK1 or p3 × Flag–CMV–RACK1 for 36 h or 48 h to knockdown or overexpress RACK1 expression, respectively. Then, the cells were infected with PRV (MOI = 1) or treated with 2^′^,3^′^‐cGAMP (1 ng/μL) for the indicated time, respectively. Then, protein levels of IRF3, p‐IRF3, RACK1, and STING in PRV‐infected PK‐15 cells were detected by western blot, respectively (A, B). Meanwhile, the levels of IRF3, p‐IRF3, RACK1, and STING in 2^′^,3^′^‐cGAMP‐treated PK‐15 cells were also detected by western blot (C,D). The levels of p‐IRF3/IRF3 protein were quantified by Image J.

**Figure:**
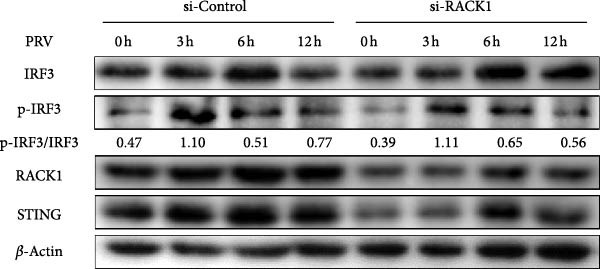
(A)

**Figure:**
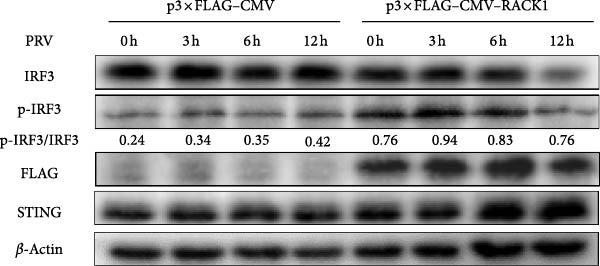
(B)

**Figure:**
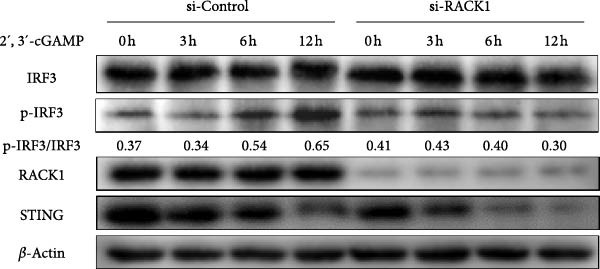
(C)

**Figure:**
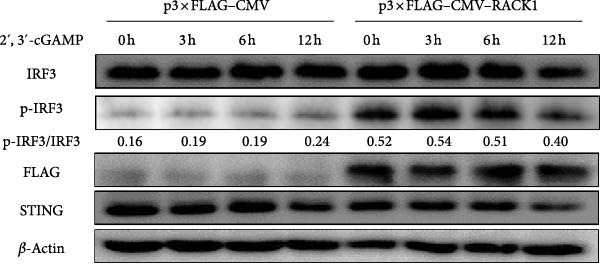
(D)

To conduct further analysis of whether RACK1 affects the phosphorylation of IRF3 through the STING pathway, 2^′^,3^′^‐cGAMP, the STING activator, was adopted to stimulate the cells instead of PRV infection. Western blot showed that the phosphorylation level of IRF3 in si‐Control group was enhanced with the extension of 2^′^,3^′^‐cGAMP treatment time, while the phosphorylation level of IRF3 in si‐RACK1 group was inhibited (Figure 4C). Furthermore, RACK1 overexpression substantially enhanced p‐IRF3 levels both in the presence and absence of 2^′^,3^′^‐cGAMP treatment (Figure 4D). Together, these results suggest that RACK1 facilitates STING‐dependent phosphorylation of IRF3 triggered by PRV infection or 2^′^,3^′^‐cGAMP treatment.

### 3.5. RACK1 Interacted With STING and Promoted Aggregation of STING Around the Golgi Apparatus

To investigate the potential interaction between RACK1 and STING, we employed AlphaFold 3 for protein–protein docking. The resulting complex was visualized in Pymol to characterize the binding interface, specifically identifying the amino acid residues from both RACK1 and STING that participate in hydrophobic contacts (Figure 5A).

Figure 5RACK1 had an interaction with STING. The image depicts the docking model of the porcine RACK1–STING complex, showing the polar contacts at the protein–protein interface. The three‐dimensional structure of porcine RACK1 (in hotpink) bound with porcine STING (in marine blue) and residues of RACK1 and STING involved in the interaction were shown in the image (A). RACK1 was found to interact with STING in HEK‐293T cells via Co‐IP assay (B, C). The colocalization of RACK1 and STING in HEK‐293T cells was visualized by confocal immunofluorescence microscopy. The scale bar in each image was 20 μm (D).

**Figure:**
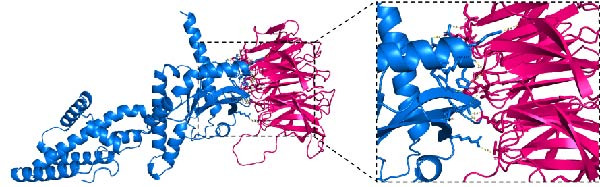
(A)

**Figure:**
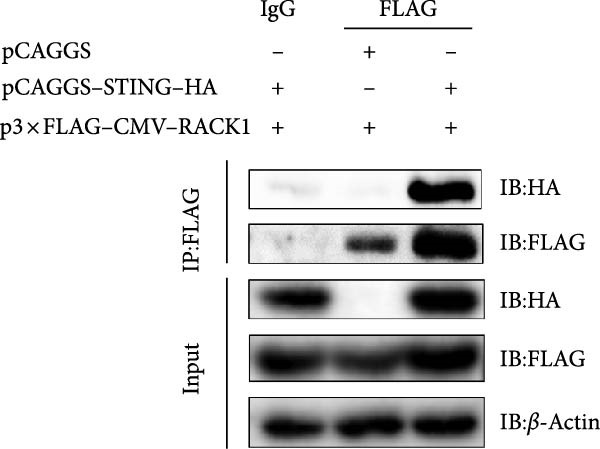
(B)

**Figure:**
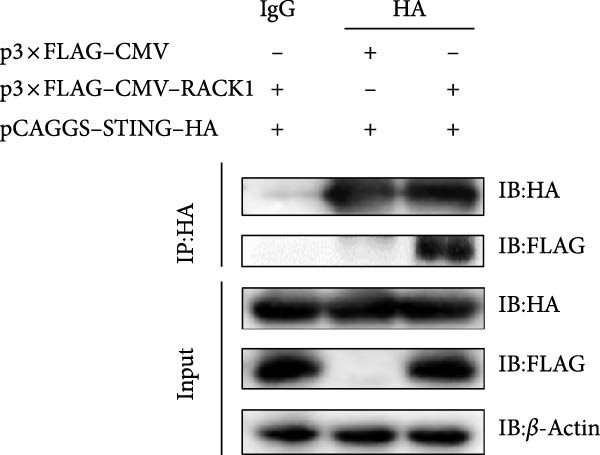
(C)

**Figure:**
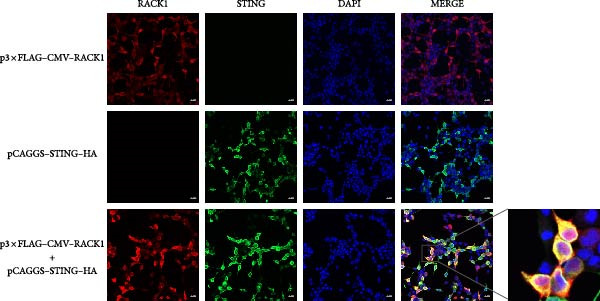
(D)

To investigate whether RACK1 can directly interact with STING, the STING expressing vector with HA tag (pCAGGS–STING–HA) and RACK1 expressing vector with Flag tag (p3 × FLAG–CMV–RACK1) were, respectively, transfected into HEK‐293T cells, and the successful overexpression of STING and RACK1 in HEK‐293T cells were confirmed (Figure S2). Then, HEK‐293T cells were cotransfected with pCAGGS‐STING‐HA and p3 × FLAG–CMV–RACK1 for 24 h. Cell lysates were incubated with anti‐Flag or anti‐HA antibodies for IP, and the immune complexes were detected by immunoblot with anti‐Flag or anti‐HA antibodies. The results showed that the distinct Co‐IP bands were detected, indicating that RACK1 had a physical interaction with STING in cells (Figure 5B,C). Meanwhile, the colocalization of RACK1 and STING in HEK‐293T cells was analyzed by laser confocal microscopy. Both RACK1 and STING were localized in the cytoplasm, and the colocalization phenomenon between RACK1 and STING was observed (Figure 5D).

Previous study showed that STING could translocated and accumulated in the Golgi after activation [30]. To further explore the influence effect of RACK1 on STING translocation, HEK‐293T cells ectopically expressing STING, or RACK1 and STING, were subjected to 2^′^,3^′^‐cGAMP stimulation or PRV infection, and the expression and cellular localization of STING was analyzed by confocal laser microscopy. The results showed that overexpression of RACK1 could promote the aggregation of STING around the Golgi apparatus in the presence or absence of 2^′^,3^′^‐cGAMP (Figure 6A). In addition, PRV infection enhanced STING expression, and RACK1 facilitated the accumulation of STING at the Golgi during PRV infection (Figure 6B). Taken together, all the data indicated that RACK1 could interact with STING and promote aggregation of STING around the Golgi apparatus.

Figure 6RACK1 promoted aggregation of STING around the Golgi apparatus. HEK‐293T cells were cotransfected with the expression plasmid of STING–HA and FLAG empty plasmid or the expression plasmids of STING‐HA and RACK1–FLAG for 24 h, respectively. Then, HEK‐293T cells were treated with 2^′^,3^′^‐cGAMP (A) or infected with PRV (B) for 3 h. After 2^′^,3^′^‐cGAMP treatment or PRV infection, the cellular localization of STING, Golgi, and nucleus were stained with HA antibody, Golgin‐97 antibody, and DAPI, respectively. The confocal immunofluorescence microscopy was then used to analyze the immunofluorescent staining of STING and Golgin‐97 in the cytoplasm. The scale bar in each image was 20 μm.

**Figure:**
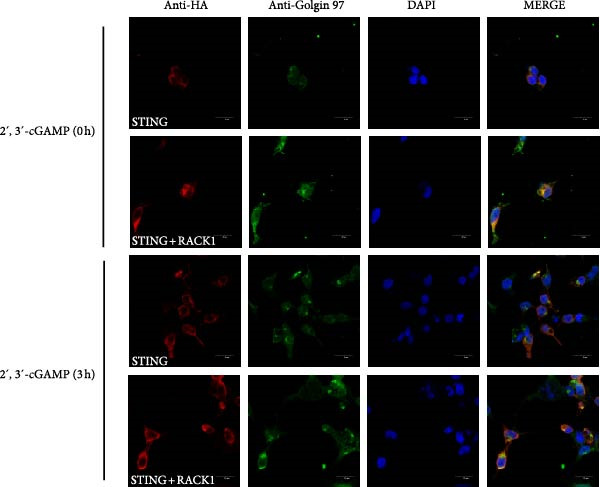
(A)

**Figure:**
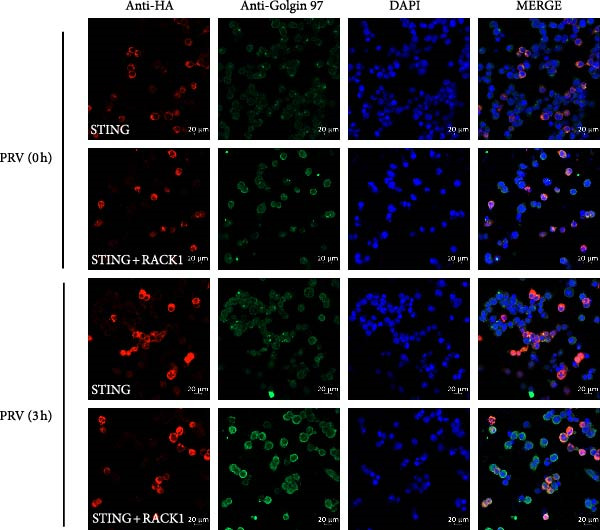
(B)

## 4. Discussion

Aujeszky’s disease caused by PRV infection has a serious impact on pigs, manifested as respiratory disease in adult pigs, reduced reproductive capacity in pregnant sows, and severe neurological symptoms in piglets [31]. In late 2011, the PRV variants emerged and occurred in pigs vaccinated with Bartha‐K61 vaccine, and then became the dominant strains in China [32, 33], which suggested that the current vaccine may not provide enough protection against PRV variants infection. Therefore, it is necessary to develop specific antiviral drugs to assist the control of viral infection. Investigation of the underlying mechanisms of host defense responses to PRV infection will provide potential strategies for the development of effective antiviral therapies.

As an intracellular scaffold protein, RACK1 has a regulatory effect on a variety of life activities in cells. Recent studies have also found that RACK1 can affect the invasion and replication of multiple viruses including LCDV, PRRSV, DENV, HCV, and CSFV, which provides a promising target for antiviral therapy [18–23]. However, the role of RACK1 in PRV infection remains unclear. In the present study, we reported an important role of RACK1 in mediating IFN‐I activation and inhibiting PRV infection. First, we found a significant inhibitory effect of PRV on the expression of RACK1, IFN‐β, ISG15, and ISG20 in cells during the late phase of viral infection, indicating a potential association between RACK1, IFN‐I pathway, and PRV infection.

Furthermore, we explored the effect of RACK1 on PRV replication by RNA interference and protein overexpression of RACK1 and found that RACK1 had strong inhibitory effects on PRV proliferation in PK‐15 cells. This result was consistent with a previous study on CSFV, which showed that RACK1 inhibited CSFV proliferation and expression of viral E2 gene in PK‐15 cells [21]. However, another study on PRRSV showed that RACK1 could promote PRRSV replication in MARC‐145 cells [18, 34]. We speculate that the opposite effect of RACK1 on viral replication may be due to the difference in virus species or the type of infected cells.

RACK1 has been implicated as a key modulator of IFN‐I signaling during viral infections. For instance, RACK1 could form a complex with protein phosphatase 2A (PP2A) to suppress phosphorylation of IRF3 and limit IFN‐I activation in response to low‐titer SeV stimulation [35]. However, the underlying mechanism by which RACK1 regulates IFN‐I pathway during PRV infection has not been reported. Here, we investigated the possible role of RACK1 in regulating activation of IFN‐I pathway during PRV infection, and found that RACK1 could significantly promote IFN‐β and several ISGs expression, suggesting that RACK1 had a capacity to activate IFN‐I signaling pathway in PK‐15 cells.

Previous studies have shown that PRV can activate the cGAS–STING–IRF3 signaling axis and promote IFN‐β expression [11]. Additionally, several host factors have been found to play important roles in regulating the cGAS–STING pathway activation during PRV infection. The heat shock protein 27 (HSP27) could degrade cGAS and attenuate cGAS‐mediated antiviral responses, thereby promoting PRV infection [36]. Inhibition of histone deacetylase 1 (HDAC1) could suppress PRV replication by activating the cGAS–STING pathway [37]. Moreover, DDX56 could target cGAS, promote cGAS–STING–induced IFN‐β expression and then inhibit PRV proliferation [38]. However, whether RACK1 plays a critical role in regulating cGAS–STING–IRF3 signaling is not determined. In the present study, we found that RACK1 could promote phosphorylation of IRF3 in the presence or absence of PRV infection. Similarly, RACK1 could also enhance phosphorylation of IRF3 induced by 2^′^,3^′^‐cGAMP (the STING agonist). Taken together, the above results indicated that RACK1 may be involved in the activation of STING–IRF3 pathway in host cells.

To further explore whether RACK1 can directly interact with STING and then activate the IFN‐I signaling pathway, the potential interaction between RACK1 and STING was predicted by protein–protein docking. Then, a Co‐IP assay confirmed that RACK1 could interact with STING, and the colocalization of RACK1 and STING in HEK‐293T cells was visualized by laser confocal microscopy. Previous study has demonstrated that STING could translocate from the endoplasmic reticulum to the Golgi after activation and then trigger IRF3–IFN‐I signaling pathway activation [30]. Interestingly, we observed via confocal microscopy that RACK1 could facilitate STING aggregation around the Golgi upon either PRV infection or 2^′^,3^′^‐cGAMP stimulation, suggesting that RACK1 could promote aggregation of STING around the Golgi apparatus and then trigger the IFN‐I pathway activation.

**Figure 7:**
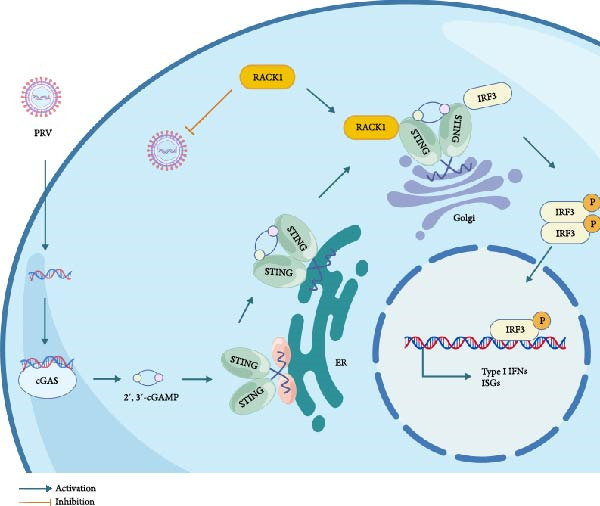
The mechanism model in this study. Our model depicts the proposed mechanism by which RACK1 restricts PRV infection. RACK1 can bind to STING, promote the aggregation of STING around the Golgi apparatus, and thereby activate the IRF3–dependent IFN‐I signaling pathway to trigger an antiviral response. The figure was created with BioGDP.com [39].

In conclusion, we demonstrated that RACK1 had the capacity to inhibit PRV infection and promote activation of IFN‐I pathway. Moreover, RACK1 interacted with STING and promoted aggregation of STING around the Golgi apparatus, thereby triggering the IRF3–dependent IFN‐I signaling pathway activation to mount an antiviral response (Figure 7). These data give new insights into the mechanisms of IFN‐I pathway activation, and facilitate our understanding of the host defense responses to PRV infection.

## Conflicts of Interest

The authors declare no conflicts of interest.

## Author Contributions

Yixuan Li and Chao Ye contributed to writing of the manuscript. Yixuan Li, Yiyu Liu, Yu Dai, and Jingyi Niu contributed to the acquisition and analysis of the data. Yixuan Li, Gang Li, Xiaoying Yu, Chao Wan, Rendong Fang, and Chao Ye contributed to the study design and data interpretation.

## Funding

This study was supported by the National Natural Science Foundation of China (Grants 32372982 and 32473027), the Natural Science Foundation of Chongqing, China (Grant CSTB2025NSCQ‐GPX0486), the National Center of Technology Innovation for Pigs (Grant NCTIP‐XD/C17), and the Chongqing Modern Agricultural Industry Technology System (Grant CQMAITS202512).

## Supporting Information

Additional supporting information can be found online in the Supporting Information section.

## Supporting information


**Supporting Information** Figure S1. Overexpression of RACK1 inhibited PRV replication in PK‐15 cells. PK‐15 cells were transfected with the empty plasmid pcDNA3.1(+) or the recombinant plasmid pcDNA3.1‐RACK1 for 48 h, respectively. And then, the overexpression effect of RACK1 in cells was verified by using western blot and qRT‐PCR, respectively (A, B). In addition, PK‐15 cells transfected with pcDNA3.1(+) or pcDNA3.1‐RACK1 were infected with PRV (MOI = 0.1) for 24 h. Western blot was used to detect the protein expression levels of RACK1 and PRV gE in cell lysates (C). The TCID50 assay was used to detect virus titer in cell supernatant (D). The statistical difference is expressed as  ^∗^ *p* < 0.05,  ^∗∗^ *p* < 0.01. Figure S2. Verification of the ectopic expression of RACK1 and STING in HEK‐293T cells. HEK‐293T cells were transfected with p3 × FLAG‐CMV or p3 × FLAG‐CMV‐RACK1 for 24 h and western blot was used to detect the protein expression levels of RACK in cell lysates with Flag antibody and RACK1 antibody, respectively (A). In addition, HEK‐293T cells were transfected with pCAGGS or pCAGGS‐STING‐HA for 24 h and western blot was used to detect the protein expression levels of STING in cell lysates with HA antibody and STING antibody, respectively (B).

## Data Availability

All data are available within the article and from the corresponding author upon reasonable request.
