# Supplementary material for: RACK1 Associates With STING to Promote Type I Interferon Activation and Inhibit Pseudorabies Virus Infection
Source: Transbound Emerg Dis. 2025 Dec 11;2025:9584967. doi: 10.1155/tbed/9584967 (PMC12697816; doi:10.1155/tbed/9584967)

**Figure S1: Overexpression of RACK1 inhibited PRV replication in PK-15 cells.** PK-15 cells were transfected with the empty plasmid pcDNA3.1 (+) or the recombinant plasmid pcDNA3.1-RACK1 for 48 h, respectively. And then, the overexpression effect of RACK1 in cells was verified by using Western blot and qRT-PCR, respectively (A-B). In addition, PK-15 cells transfected with pcDNA3.1 (+) or pcDNA3.1-RACK1 were infected with PRV (MOI=0.1) for 24 h. Western blot was used to detect the protein expression levels of RACK1 and PRV gE in cell lysates (C). The TCID_50_ assay was used to detect virus titer in cell supernatant (D). The statistical difference is expressed as **p* < 0.05, ***p* < 0.01.


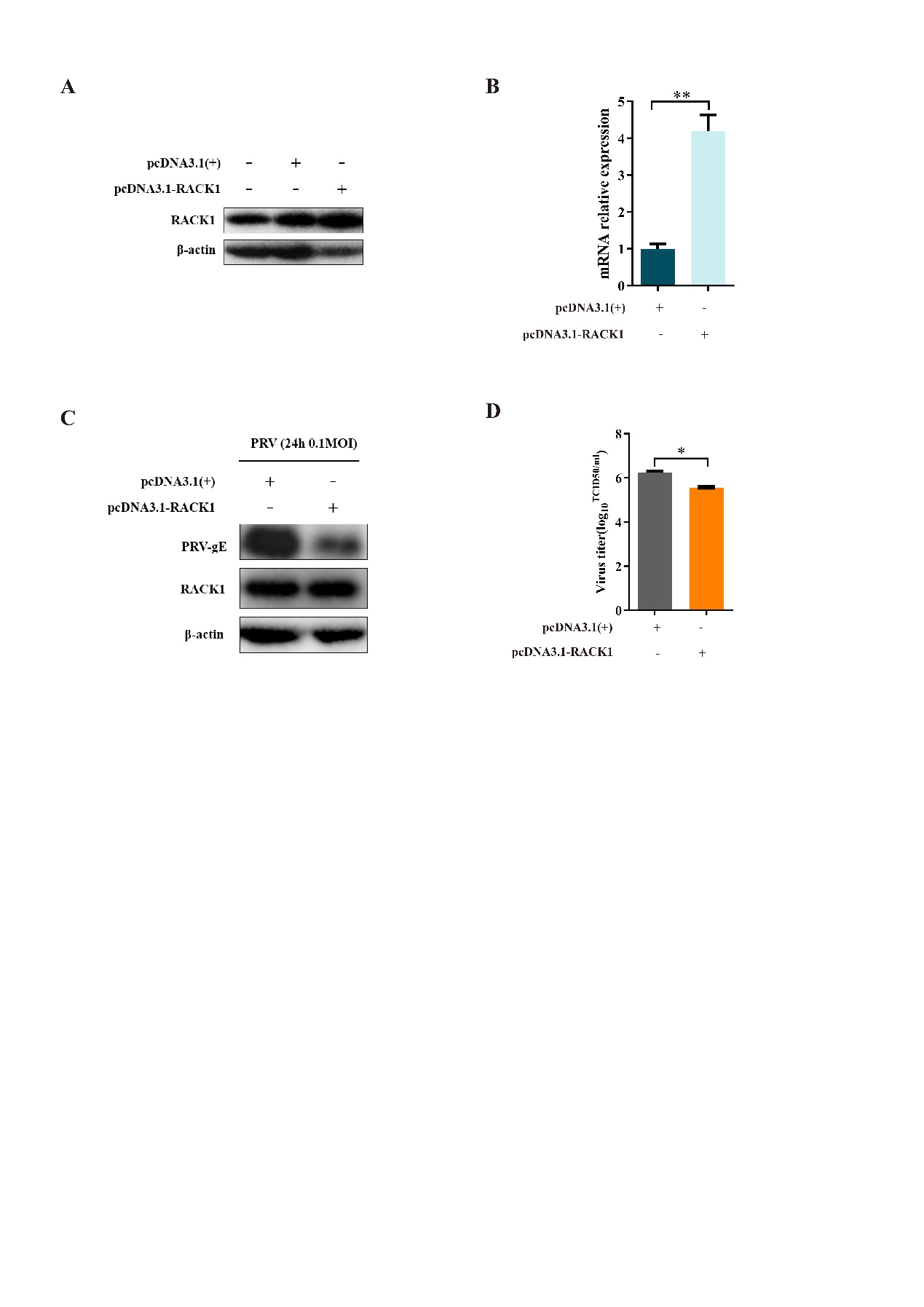


**Figure S2: Verification of the ectopic expression of RACK1 and STING in** **HEK-293T cells.** HEK-293T cells were transfected with p3×FLAG-CMV or p3×FLAG-CMV-RACK1 for 24 h and Western blot was used to detect the protein expression levels of RACK in cell lysates with Flag antibody and RACK1 antibody, respectively (A). In addition, HEK-293T cells were transfected with pCAGGS or pCAGGS-STING-HA for 24 h and Western blot was used to detect the protein expression levels of STING in cell lysates with HA antibody and STING antibody, respectively (B).


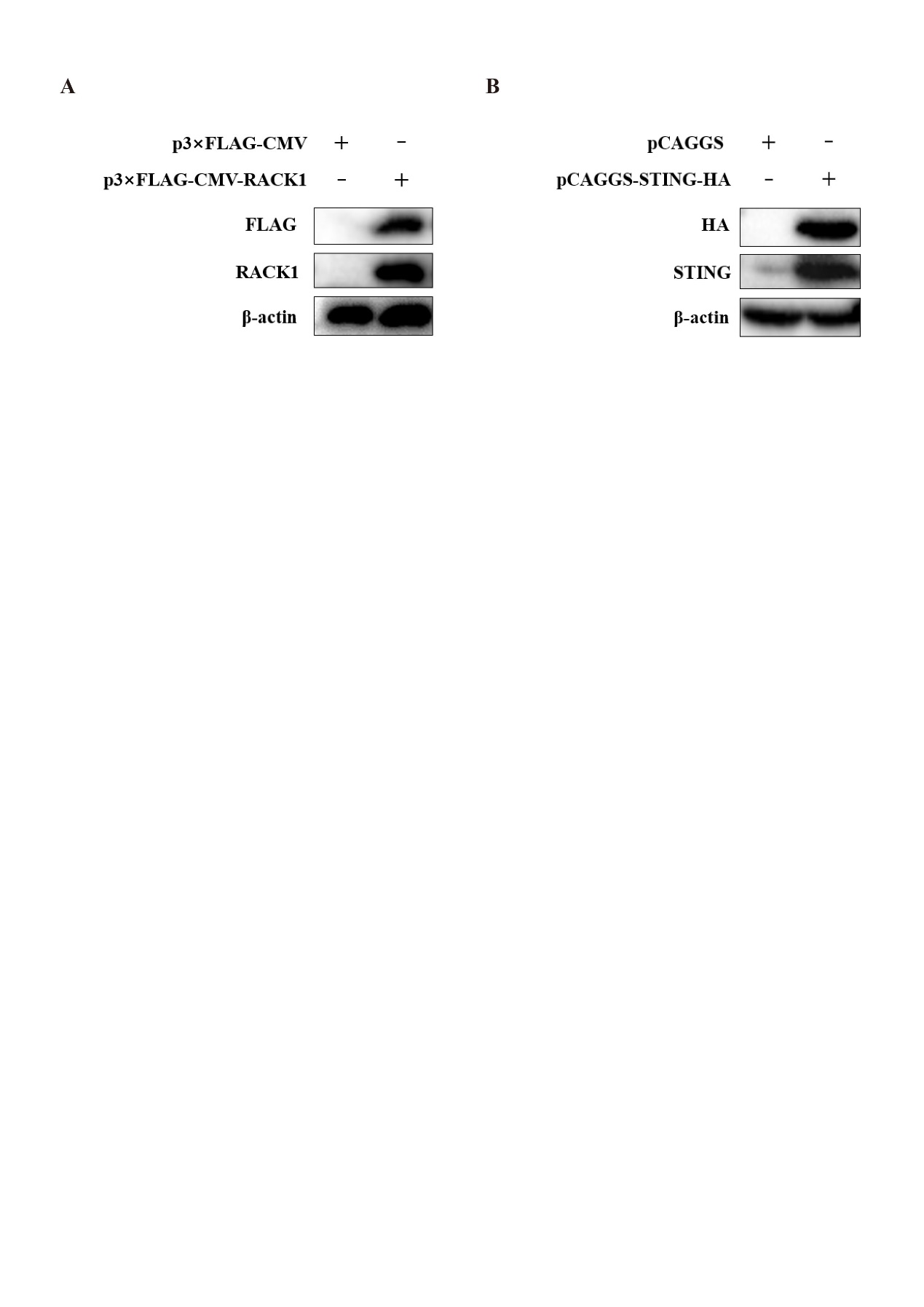

Supplement: Supplementary file 1 — Supporting Information Figure S1. Overexpression of RACK1 inhibited PRV replication in PK‐15 cells. PK‐15 cells were transfected with the empty plasmid pcDNA3.1(+) or the recombinant plasmid pcDNA3.1‐RACK1 for 48 h, respectively. And then, the overexpression effect of RACK1 in cells was verified by using western blot and qRT‐PCR, respectively (A, B). In addition, PK‐15 cells transfected with pcDNA3.1(+) or pcDNA3.1‐RACK1 were infected with PRV (MOI = 0.1) for 24 h. Western blot was used to detect the protein expression levels of RACK1 and PRV gE in cell lysates (C). The TCID50 assay was used to detect virus titer in cell supernatant (D). The statistical difference is expressed as ∗ p < 0.05, ∗∗ p < 0.01. Figure S2. Verification of the ectopic expression of RACK1 and STING in HEK‐293T cells. HEK‐293T cells were transfected with p3 × FLAG‐CMV or p3 × FLAG‐CMV‐RACK1 for 24 h and western blot was used to detect the protein expression levels of RACK in cell lysates with Flag antibody and RACK1 antibody, respectively (A). In addition, HEK‐293T cells were transfected with pCAGGS or pCAGGS‐STING‐HA for 24 h and western blot was used to detect the protein expression levels of STING in cell lysates with HA antibody and STING antibody, respectively (B). [file TBED-2025-9584967-s001.docx]
